# Is Virtual Fencing an Effective Way of Enclosing Cattle? Personality, Herd Behaviour and Welfare

**DOI:** 10.3390/ani12070842

**Published:** 2022-03-27

**Authors:** Magnus Fjord Aaser, Søren Krabbe Staahltoft, Andreas Hein Korsgaard, Adam Trige-Esbensen, Aage Kristian Olsen Alstrup, Christian Sonne, Cino Pertoldi, Dan Bruhn, John Frikke, Anne Cathrine Linder

**Affiliations:** 1 Department of Chemistry and Bioscience—Section of Bioscience and Engineering, Aalborg University, Fredrik Bajers Vej 7H, 9220 Aalborg, Denmark; maaser20@student.aau.dk (M.F.A.); sstaah20@student.aau.dk (S.K.S.); akorsg20@student.aau.dk (A.H.K.); atrige20@student.aau.dk (A.T.-E.); cp@bio.aau.dk (C.P.); db@bio.aau.dk (D.B.); 2 Department of Nuclear Medicine and PET, Aarhus University Hospital, Palle Juul-Jensens Boulevard 165, 8200 Aarhus, Denmark; aage.olsen@clin.au.dk; 3 Department of Clinical Medicine, Aarhus University, Palle Juul-Jensens Boulevard 165, 8200 Aarhus, Denmark; 4 Department of Ecoscience, Aarhus University, Frederiksborgvej 399, 4000 Roskilde, Denmark; cs@ecos.au.dk; 5 Aalborg Zoo, Mølleparkvej 63, 9000 Aalborg, Denmark; 6 Skagen Bird Observatory, Fyrvej 36, 9990 Skagen, Denmark; 7 Wadden Sea National Park, Havnebyvej 30, 6792 Rømø, Denmark; jofri@danmarksnationalparker.dk

**Keywords:** virtual fencing, Nofence©, Angus cattle, animal welfare, animal behaviour, animal personality, nature conservation

## Abstract

**Simple Summary:**

Virtual fences provide boundaries without physical barriers. Virtual fencing systems utilise a collar and GPS technology for tracking animals and delivering audio warnings and electric impulses to the animals when approaching the designated virtual boundary. These GPS-based fencing systems have the potential to improve grazing management. This article examines the use of the Nofence© virtual fencing system to keep a group of twelve Angus cows within a virtual enclosure without compromising animal welfare. Within 139 days the cows had learned to respond to the auditory warnings, thus, respecting the virtual boundaries. The virtual fence was generally successful in keeping the cows within the virtual enclosure. The virtual enclosure was expanded and subsequently made smaller several times, and the animals did not show significant issues adapting to the new border placement. The cattle did not express any significant changes in their behaviour upon receiving an electrical impulse from the collar. However, they did display inter-individual differences, indicating that the personality of the cows should be taken into account when selecting animals for placement in virtual enclosures. The cows also reacted to herd mates receiving electric impulses showing that they are influenced by their herd mates.

**Abstract:**

In modern nature conservation and rewilding there is a need for controlling the movements of large grazers in extensively managed areas. The inflexibility of physical fencing can be a limitation in nature management, and the physical boundaries created by physical fencing can have detrimental effects on wildlife. Virtual fencing systems provide boundaries without physical structures. These systems utilise collars with GPS technology to track animals and deliver auditory or electric cues to encourage the animals to stay within the predefined boundaries. This study aims to assess the use of virtual fencing (Nofence©) to keep twelve Angus cows (*Bos taurus*) within a virtual enclosure without compromising their welfare. As such, the study examines inter-individual differences between the cows as well as their herd behaviour, when reacting and learning to respond appropriately to virtual fencing. Moreover, the activity of the cows was used as an indicator of welfare. The virtual fencing was successful in keeping the herd within the designated area. Moreover, the cattle learned to avoid the virtual border and respond to auditory cues, where the cows received significantly more auditory warning and electric impulses per week throughout the first 14 days than the remaining 125 days (*p* < 0.001). The cows were found to express both inter-individual differences (*p* < 0.001) and herd behaviour. The cattle did not express any significant changes in their activity upon receiving an electrical impulse from the collar. Thus, indicating that there were little to no acute welfare implications associated with the use of virtual fencing in this study. This study clearly supports the potential for virtual fencing as a viable alternative to physical electric fencing. However, it also shows that both individual differences in personality and herd structure should be considered when selecting individuals for virtual fencing.

## 1. Introduction

In recent years, nature conservationists have become aware of the beneficial effects of rewilding, with large organisations such as the European Union expressing interest in the subject [1]. Rewilding utilises large grazers in large areas to achieve the desired effects on the composition of vegetation in the landscape, increasing the need for large peripheral fencing. Physical barriers, such as those created by physical electric fencing, can have detrimental effects on wildlife [2,3]. Moreover, physical fencing cannot be readily adapted to changing circumstances. The inflexibility of physical fencing is often described as a limitation in nature management, e.g., when dealing with seasonal changes in vegetation, or periods when animals need to be excluded from environmentally-sensitive areas [4,5]. Virtual fencing has, therefore, been developed as an alternative to physical fencing. This type of fencing using GPS technology has been shown to be an effective way of keeping animals within an area [6,7,8,9,10]. This could prove to be a flexible fencing option for extensive grazing of large areas.

Virtual fencing works by attaching a collar to each animal that can administer auditory warnings ( 82 dB, 1 m) and low energy electric impulses ( 0.2 J, 3 kV, 1.0 s). However, this is illegal in a number of European countries including Denmark, under current regulations. Moreover, animal welfare has been shown to be a concern for the public when it comes to implementing virtual fencing technology [11]. Thus, to determine whether virtual fencing is suitable for use in nature conservation, it has to be ensured, that welfare is not compromised. When referring to animal welfare in this paper the term is mainly associated with the basic health and functioning of the animals in relation to the animals’ behaviour. It is, therefore, crucial that the animals are capable of learning to respond to audio cues to avoid unnecessary stress responses.

Previous studies have shown that animal welfare in virtual enclosures is comparable to physical electric fencing, and that cattle and sheep can learn to respond to audio cues and thereby avoid electric impulses [5,12,13,14,15,16,17,18]. Thus, suggesting that virtual fencing does not have a negative impact on animal welfare compared to current practices, i.e., physical electric fencing. However, several of these studies investigated the cattle collectively as a group but noted a high inter-individual variance, thus, calling for further research accounting for inter-individual differences [8,12,14,19,20]. Many factors can influence this intra-specific variation in behaviour, but one important factor to consider when choosing individuals for virtual fencing could be the expression of personality [21,22]. Animal personality can be defined as individual differences in behavioural traits that are consistent over time and across contexts [23,24]. Another apparent problem with pooling individuals in welfare assessments is the fact that cattle are social animals living together in herds. Therefore, they cannot be considered as completely independent units as they will experience socially facilitated learning, as well as possibly reacting to a herd mate being exposed to a stressful situation, such as receiving an electric impulse [20,25,26,27]. Thus, there is a need for an assessment of individual cow welfare in virtual enclosures and their individual learning ability to respond appropriately to the auditory cues [20,28].

Virtual fencing could provide useful in the establishment of the new nature national parks that are underway. Enclosing these large areas with physical fencing has been subject to some public opposition. Using virtual fencing eliminates the presence of a physical barrier, thus, eliminating many of the concerns associated with physical fencing. However, the current regulations in Denmark prohibit the use of virtual fencing, due to the unfamiliarity of such systems and their effect on animal welfare. Therefore, this study aims to assess the use of the Nofence© system to keep a group of cattle within a virtual enclosure with minimal welfare implications, i.e., without impairing the cattle’s basic health and functioning. This was achieved by studying the individual behaviour of twelve Angus cows (*Bos taurus*) in a virtual enclosure wearing Nofence© collars, along with the behaviour of the entire herd collectively. It was hypothesised that the Nofence© virtual fencing system would be successful in keeping the cows within the virtual enclosure. Furthermore, the cows were hypothesised to learn to respond appropriately to the virtual fence, i.e., reacting to the audio cues alone and receiving fewer electric impulses. In order to thoroughly assess the use of the virtual fencing system in practice, this study also explored individual and herd factors affecting learning capacity. It was hypothesised that the cows in this experiment would express inter-individual differences. The cows were also hypothesised to express herd behaviour by reacting to electric impulses received by herd mates. Lastly, the activity of the cattle was utilised as an indicator of welfare. Here, it was hypothesised that the general level of activity of the cows would not differ immediately before and after receiving an electric impulse, indicating no effect on their basic health and functioning.

## 2. Materials and Methods

### 2.1. Animals and Location

**Figure 1:**
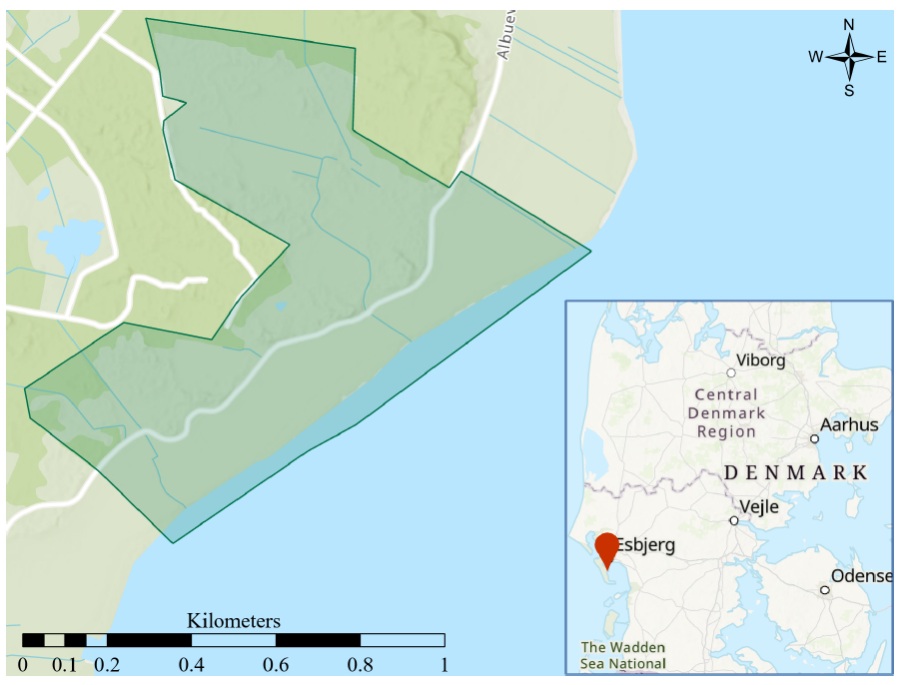
The 65 ha study area on Fanø in the southwest of Denmark. The dark green area marks the area encompassing the largest iteration of the virtual enclosure. The coordinates of the upper left corner of the map frame are 8${}^{\circ }$26${}^{\prime }$33${}^{\prime \prime }$ E 55${}^{\circ }$24${}^{\prime }$28${}^{\prime \prime }$ N.

The study took place on the east coast of the island of Fanø in the southwest of Denmark (Figure 1). The area consisted of 65 ha of coastal meadows to the east and a dune landscape with both dry (heath) and wet parts, with scattered vegetation of trees and bushes to the west. As a sort of border between the two habitat types, a gravel road runs through the study area from north to south, and further two summer residences are nestled on two small dune tops (Figure 1). The animals had access to lush grass close to the waterline and dry areas for resting further inland. The experiment was carried out utilising twelve Angus cows aged between four and nine years at the start of the experiment. All cows were pregnant and calved during the experiment. The cows were used to traditional physical electric fencing, but not familiar with virtual fencing prior to the study.

### 2.2. Virtual Fencing System

All data in this experiment was collected using collars developed by the company Nofence© (https://www.nofence.no/en/, accessed on 25 March 2022) fitted around the neck of the animals (Table 1) (Appendix A) [29]. The collars weighed 1446 g and were composed of a silicone strap, placed on top of the animals’ neck, connected to two chains that held up a box suspended below the animals’ neck, containing the battery, two small solar panels and a GPS receiver. The collars measured the position, heading and speed of the animals. An animal’s position is triangulated using the GNSS positioning system (GPS and GLONASS). Heading and speed are continuously calculated from the last two positions of the animal. Each collar had a unique serial number that was included in every message sent by the collar, which remained constant during the experiment. All cows except one, wore the same collar during the entire experiment. Data points from the animal that had a change in collar were given the same serial number during analysis. This means that each cow had a unique serial number used to reference the animal.

A virtual boundary was specified, and the collar then used an animal’s GPS position to determine if the animal was approaching the virtual boundary (Figure 1). If the animal was found to be approaching the boundary the collar would emit a series of warning sounds, consisting of multiple 82 dB tones increasing in pitch for a duration of 5–20 s, based on whether the animal continued to ignore the warning or turned around. If the animal continued and was in risk of crossing the virtual border, as determined by position, heading, and speed, it would receive an electric impulse of 0.2 J at 3 kV for one second. If the animal slowed down, changed heading, or stopped, the animal did not receive an electric impulse. The animal would continue to receive electric impulses if it did not return to the virtual enclosure up to a maximum of three impulses. If the animal had received three impulses and had not returned to the enclosure the collar would send a notification to the owner, that the animal had escaped. The collar would continue to monitor the animal’s position, but the animal would not receive further warnings or electric impulses. If, however, the animal returned to the virtual enclosure the collar returned to normal function and would again administer both warnings and electric impulses when the animal approached the virtual fence. These settings were predetermined by Nofence©.

The experiment began on 29 May 2021 and data collection for this study concluded on 14 October 2021. During the experiment the collars continuously monitored the cows’ GPS positions, but only logged GPS data every 15 min and every time a warning or electric impulse was given. Furthermore, the collar recorded the cows’ movement and logged their activity every 30 min. The activity of the animals was measured using an accelerometer as a unitless value, as it was originally developed for sheep. Information about warnings and electric impulses were recorded and transmitted at the time of the event (Table 1).

### 2.3. Experimental Protocol

On 28 May 2021, twelve cows were placed in an enclosure of about 6.5 hectares, with all four sides being physically electrically fenced. After two days the southern fence line was removed and replaced by a virtual border and the experiment began (Appendix B, Version L.1). This border was then moved about 20 m further south after an additional six days (Appendix B, Version L.2). Then, the border was moved further 20 m twice after three days each (Appendix B Versions L.3 and L.4). On day 14 of the experiment the remaining three sides of the electric fence were removed and the cows were allowed to move freely within a completely virtual enclosure of 35 hectares (Appendix B, Version 1.1). In the following month and a half the enclosure was progressively expanded to 49 hectares until day 62 of the experiment when the cows were then limited to an area of 28 hectares for twelve days and then 15 hectares the following 14 days (Appendix B, Versions 1.1–2.1). After having been limited to a smaller area, the enclosure was then re-expanded to encompass 65 hectares (Appendix B, Versions 2.2–2.6). During the entire experiment, all animals were provided with pasture water pumps but no supplementary food was given. The experiment concluded after 139 days.

### 2.4. Data and Statistical Analysis

Data was collected as different message types (Table 1). *Poll* messages were sent periodically every 15 min and contain the collar status. The collar status includes the time, the cow’s position (latitude and longitude), and the battery voltage in centivolts. *Seq* messages describe the solar charge gathered in the last 30 min and the number of “steps” the cow has moved in the last 30 min. The number of steps was given as a unitless value, as the accelerometer was originally developed for sheep. *Warning* messages were sent when the collar has finished playing a warning sound, either because the cow has turned around, i.e., understanding the warning sound, or because the cow has received an electric pulse. *Warning* messages also contain the duration of the warning sound in milliseconds. *Zap* messages were sent when the collar gives of an electric pulse. One of the cows had its collar changed during the experiment, and for that reason, one day of data was removed for that single individual. No other problems with the collars were discovered.

In total 238,899 messages were recorded by the GPS collars over the course of the 139-day experiment. Of these messages 157,120 were *poll* messages, 79,633 were *seq* messages, 1949 were *warning* messages, and 197 were *zap* messages. For statistical analysis, data was subdivided by message type and the individual from which the data was collected. The data was further divided into three separate periods. *The learning period* being from the beginning of the experiment until day 14, where the remaining physical electric fence was removed, enlarging the enclosure, which now only consisted of virtual borders. *The first period* within the complete virtual enclosure, thus, began on day 15 and lasted until, but not including day 75 where the enclosure size was considerably reduced. *The second period* consequently lasted from day 75 until the end of the experiment on day 139. All data was plotted and sorted in QGIS [30] and all statistical analyses were carried out in R version 4.1.2 [31]. Non-parametric tests were used in the statistical analysis, as the data was not normally distributed.

#### 2.4.1. Distribution within the Virtual Enclosure

In order to evaluate if the Nofence© system was successful in keeping the cows within the virtual enclosure, a heatmap was created for each version of the virtual enclosure, thus, portraying the cows’ distribution in relation to the virtual borders throughout the study (Appendix C).

#### 2.4.2. Learning Ability

To determine whether the cows were able to learn to respond appropriately to virtual fencing, thus, receiving fewer warnings and electric impulses with time, the cumulative number of warnings was calculated for each individual and assessed in relation to changes in the virtual border. Differences between the three periods were investigated by comparing the average number of warnings received by the individuals per week for the respective periods using the Mann-Whitney U test. The average number of electric impulses received by the individuals per week was also compared across the three periods with the Mann-Whitney U test.

#### 2.4.3. Inter-Individual Differences

In order to assess any inter-individual differences between the cows, the individuals were compared in regard to the number of auditory warnings and electric impulses they received. The Kruskal Wallis test was used to determine if there was a significant difference between individuals in regard to the number of warnings and electric impulses received, respectively. These individual differences were further investigated by conducting pairwise comparisons between all individuals using the Mann-Whitney U Test with Bonferroni’s correction. Furthermore, the relationship between the number of warnings and number of electric impulses received by the individuals throughout the experiment was tested with Spearman’s rank correlation ($r_{s}$). This relationship was assessed for the entire experiment and each of the three periods, respectively. Moreover, the number of warnings that each individual received within a given period was compared to that of the other two periods with Spearman’s rank correlation ($r_{s}$). Likewise, the number of electric impulses that each individual received within a given period was also compared to that of the other two periods with Spearman’s rank correlation ($r_{s}$).

#### 2.4.4. Herd Behaviour

To assess the herd behaviour of the cows, i.e., cows reacting to a herd mate being exposed to a stressful situation, the reaction of each individual cow was investigated in response to a herd mate receiving an electric impulse. Every time a collar gave an electric impulse to any cow (except following a social panic reaction), the distance from each individual to the virtual border was measured 15, 30, 45 and 60 min prior to the electric impulse and 15, 30, 45 and 60 min after the electric impulse. The difference in median distance to the virtual border, before and after an electric impulse, was tested for each individual using the Mann-Whitney test as well as for the median distance of the herd. This was done separately for each of the three periods.

#### 2.4.5. Reactions to Electric Impulse

To investigate whether the activity level of the cows differed after receiving an electric impulse, the total activity of an individual was recorded two hours prior to and two hours following an electric impulse, respectively. Differences in activity before and after receiving an electric impulse were assessed using the Mann-Whitney test. If several electric impulses were administered to an individual in close succession, the activity was only measured two hours before and after the first electric impulse. Additionally, a single occurrence of an individual receiving an electric impulse was removed from the data set as it was not possible to get a representative measure of activity due to the collar failing to record the activity data for a three hour period.

## 3. Results

### 3.1. Interactions with the Virtual Border

**Figure 2:**
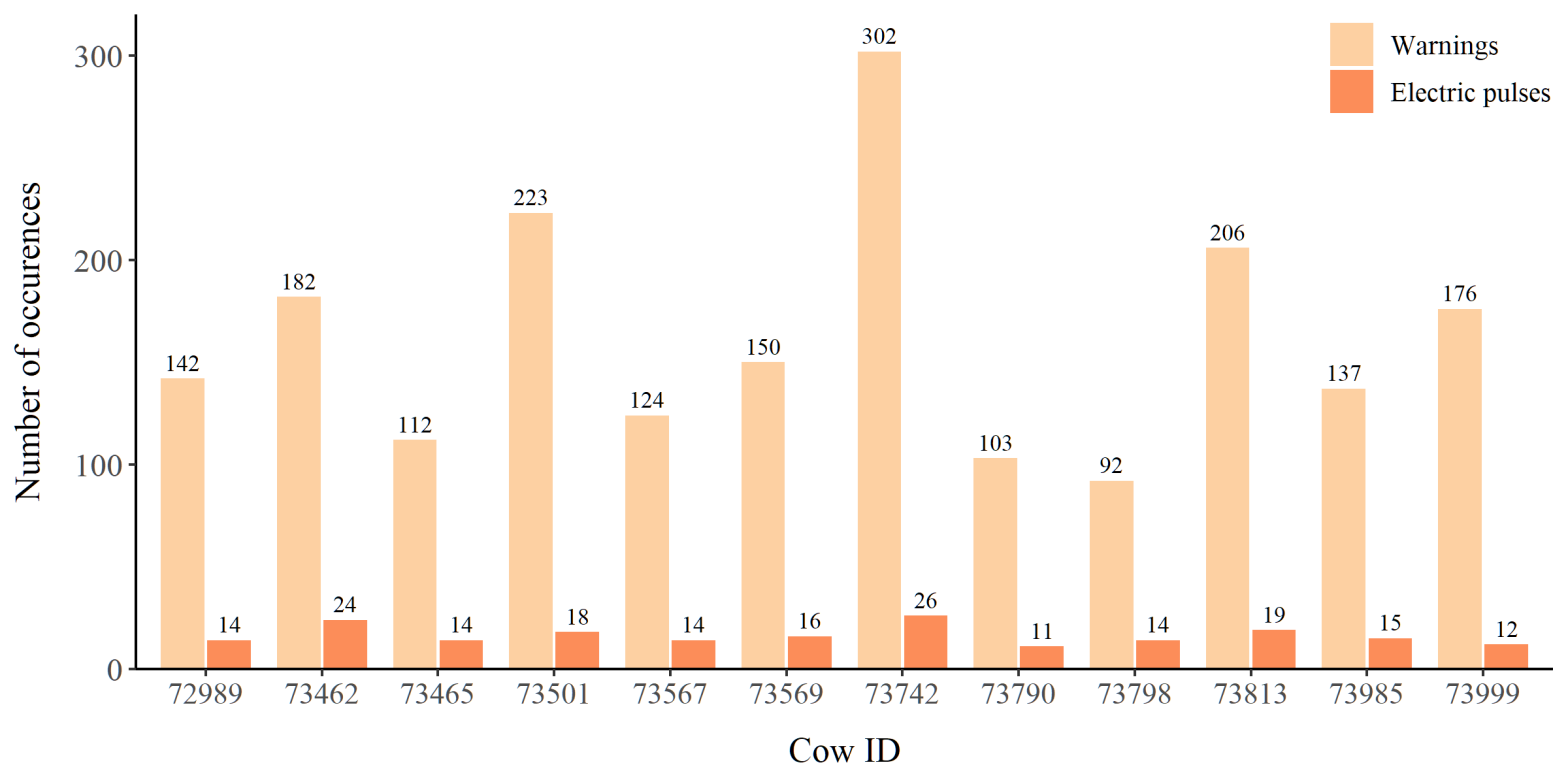
The number of auditory warnings and electric impulses that each individual received throughout the 139-day experiment.

The twelve cows received 197 electric impulses with each cow receiving between 11 and 26 electric impulses. The cows received 1949 warnings with each cow receiving between 92 and 302 warnings (Figure 2).

The virtual fence successfully kept the cows within the virtual enclosure for the majority of the experiment (Appendix C). On four separate occasions, one cow escaped the enclosure alone, with one other breakout involving three cows. Furthermore, twice all twelve cows escaped and once eight cows escaped due to a social panic reaction. The first time was during the first day of the experiment when the cows experienced the electric impulse for the first time. The second time the virtual border had been placed immediately on the other side of a ditch from the cows, which meant that when the cows tried to cross the ditch they received an electric impulse in the middle of crossing, meaning their only option was to continue forward and out of the enclosure. The border was subsequently moved to avoid further incidents. The final stampede occurred when a journalist flew a drone in low altitude towards and directly above the herd. All escaped cows were brought back to the enclosure within short time. Note that these three social panic reactions account for 104 of the electric impulses received throughout the study and 220 of the warnings received. However, these social panic reactions generally did not affect the results (Appendix F).

### 3.2. Learning Ability

**Figure 3:**
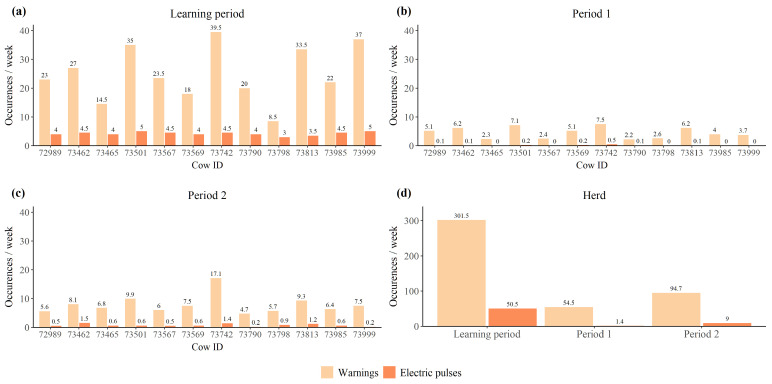
Average number of auditory warnings and electric impulses received per week by (**a**) each individual in *the learning period*, (**b**) each individual in *period 1*, (**c**) each individual in *period 2*, and (**d**) by the entire herd during each of the three periods.

The cows received significantly more auditory warnings and electric impulses per week during the 14 day *learning period* than during the other two periods (*p* < 0.001) (Figure 3). The cows also received significantly more warnings and electric impulses in *period 2* compared to *period 1* (*p* < 0.01) (Figure 3). Moreover, the ratio between the number of electric impulses and the number of auditory warnings was highest in *the learning period* for most of the individuals and all individuals had the lowest impulse to warning ratio in *period 1*.

**Figure 4:**
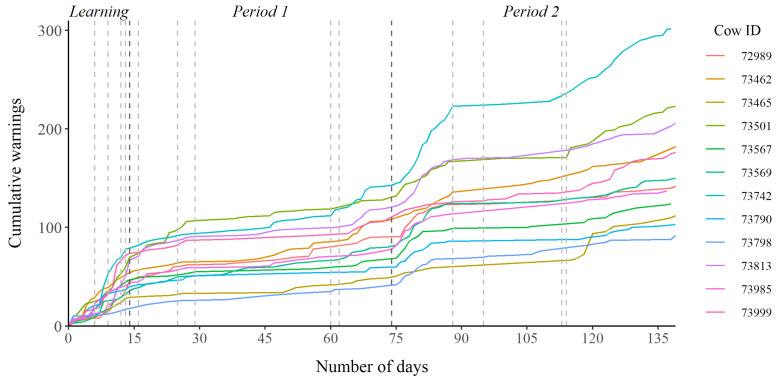
Cumulative number of warnings received by each cow during the three periods. The vertical lines in the plot mark any significant change in the virtual enclosure, where the darker lines indicate the division of the three periods.

When assessing the cumulative warnings each cow received during the three periods, the warning frequency was largest during the beginning of each period and the frequency began to plateau with time (Figure 4). However, in *period 2* there was an increase in warning frequency after the virtual border was changed on day 113. This change in the virtual border allowed the cows to move freely on either side of a gravel road but not on the road itself. This meant the cows had to follow the border some distance into the enclosure to reach a point of free passage across the road, resulting in a disproportionate number of warnings being given in the last period of the experiment.

### 3.3. Inter-Individual Differences

There was a positive correlation between the number of warnings and electric impulses an individual received over the entire period of the experiment ($r_{s}=0.762$ **) (Appendix D). There was also a positive correlation between the number of warnings an individual cow received in *the learning period* and the number of warnings received in *period 1* ($r_{s}=0.660$ *) and *period 2* ($r_{s}=0.687$ *), respectively (Appendix D). Moreover, the number of warnings an individual received in *period 1* and the number of warnings received in *period 2* were also positively correlated ($r_{s}=0.803$ **). Furthermore, the individuals significantly differed from one another in the number of warnings and electric impulses they received (*p* < 0.001) (Appendix E).

### 3.4. Herd Behaviour

**Figure 5:**
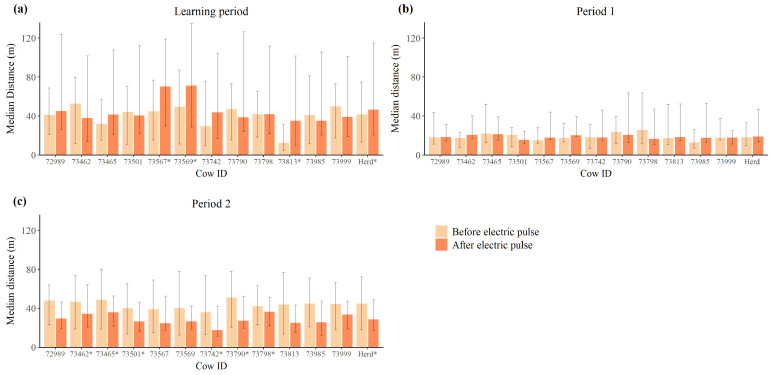
Median distance to the virtual border of each individual cow and the herd collectively, before and after any one individual received an electric impulse during (**a**) *the learning period* (n=3077), (**b**) *period 1* (n=402), and (**c**) *period 2* (n=4831). Error bars represent the interquartile range. An asterisk next to the serial number denotes statistical significance (*p* < 0.05).

During *the learning period*, the cows’ distance to the virtual border was significantly greater after any one individual received an electric impulse for three of the cows, and for the herd as a whole (*p* < 0.05) (Figure 5a). In the *period 1* of the experiment no significant differences were found in regard to the cows’ distance to the virtual border before and after any one individual received an electric impulse (Figure 5b). During *period 2*, the median distance to the virtual border was significantly smaller after any one individual received an electric impulse for six of the twelve cows and for the herd as a whole (*p* < 0.05) (Figure 5c).

### 3.5. Reactions to Electric Impulse

**Figure 6:**
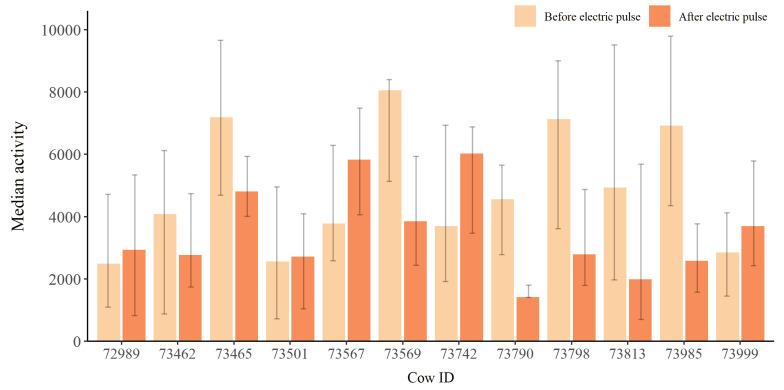
Median activity of each individual two hours before and after receiving an electric impulse (n=202). Error bars represent the interquartile range.

There were no significant changes in the cows’ activity before and after receiving an electric impulse (Figure 6).

## 4. Discussion

### 4.1. Interactions with the Virtual Border

In this study, virtual fencing was successful in keeping the herd of cattle within the specified area for the vast majority of the time, with only four separate breakouts across the entire 139-day period not explained by unfortunate circumstances such as poor fence placement or low flying drones. These results are in accordance with previous studies and underline the potential for virtual fencing in cattle management [7,8,9,10,32]. Interestingly, the cows spent most of their time on either lush areas for grazing or higher areas for resting, and did not appear to have much interest in testing the border or breaking out of the enclosure (Appendix C). It was observed, however, that the herd spent more time grazing the fresh, not previously grazed, areas whenever the virtual border was moved out, especially when kept within a smaller area. This could suggest that one should be wary of keeping the cattle in areas with limited feed availability. However, previous studies have found that it is possible to exclude cattle from reaching an area of greater feed availability to some extent [14,19]. However, this study mainly investigated the cattle individually and not as a herd, warranting further studies of whether herding behaviour can override the individuals’ hesitation to cross the virtual border in the case of low feed availability. This has previously proven to be an issue when testing similar virtual fencing systems on sheep [16,33].

In *the learning period*, the distance to the virtual border was significantly larger after any one individual received an electric impulse than prior to the impulse. This was the case for the herd as a whole and for three of the individual cows (Figure 5a). Interestingly, in *period 2* the distance to the border was significantly smaller after an individual received an electric impulse than before, both for the herd as a whole, and for six out of the twelve individuals (Figure 5c). This may be due to the cows learning the association between the auditory warning and the virtual border, thus, increasing their reliance on these cues to ensure that they remain within the virtual boundary rather than relying upon the response of their conspecifics. Nonetheless, an individual receiving an electric impulse clearly solicited a social response in its herd mates, as suggested by previous studies and supporting the initial hypothesis [25,27].

In this study there were no significant differences in the activity of the cows after receiving an electric impulse, i.e., receiving an electric impulse did not have a long term effect on the cows’ activity level. Thus, indicating that the virtual fencing system did not negatively impact the cows’ welfare, based on measures associated with the behaviour of the cows. Campbell et al. [6] similarly found a minimal impact of the virtual border on cattle behaviour. However, McSweeney et al. [17] found cattle to express some signs of stress when kept under virtual fencing. It can, therefore, not be dismissed that under some circumstances virtual fencing may have a negative effect on welfare. Such an effect on welfare could be dependent on the intensity of the electric pulse, where a more intense electric impulse would likely result in a stronger behavioural response. Further studies may provide insight into under which conditions attribute to ensuring optimal animal welfare when using virtual fencing systems. Furthermore, this study only investigated the long term effect of an electric impulse on the cows’ behaviour. It may, therefore, be relevant to also investigate the acute behavioural effect of receiving an electric impulse.

### 4.2. Learning Ability

In this study there was a positive correlation between the number of warnings and the number of electric impulses received. As such, this study suggests that an individual receiving a higher number of warnings will also receive a higher number of electric impulses. Likewise, a decrease in the number of warnings received over time will result in the individual receiving fewer electric impulses, i.e., fewer stressful events. However, the results also indicated that the ratio between the number of electric impulses and the number of auditory warnings decreased with time, showing that this relationship between the number of warnings and impulses may be subject to change as the cows improve their response to the auditory warnings alone. Thus, as expected and in accordance with previous studies, the results of this study clearly showed that the cows learned to respond correctly to the virtual fencing system [8,14]. As the number of warnings and thereby electric impulses decreased rapidly over time when the cows learned to respond appropriately to the auditory cues, enabling to avoid the virtual border, the long term welfare implications are expected to be minimal, as has also been suggested by multiple other studies [5,13,18,28,34].

It is obvious, that the cows in this study learned to avoid the virtual border, receiving fewer warnings and electric impulses over time. This corresponds well with findings in previous studies of cattle [14,15,17,19,25,35]. In the context of nature conservation and rewilding, where large grazers are meant to be kept in large areas, these results are promising when considering virtual fencing as a replacement of traditional electric fencing. This is in line with previous studies suggesting virtual fencing could be a good alternative in extensive grazing situations, such as nature conservation [2,32]. The fact that the cows seemingly have to go through a new “exploratory” phase, however, when being restricted to a far smaller area from one day to the next, suggests that such sudden changes in the enclosure should be done with precaution, as it could cause unnecessary stress to the animals. However, another study found no issue when limiting a small heard of cattle to a smaller area, the herd quickly moving into the new allowed area with no significant change in behaviour [6]. Nonetheless, future studies should pay more attention to the placement of the virtual border. A few times during the experiment the virtual border was placed sub-optimally, such as right on the other side of a ditch, or on both sides of a road such that only the road was inaccessible. This caused a disproportionately large amount of warnings and electric impulses to be delivered in these areas. Thus, future studies should also investigate optimal placement of the virtual border.

### 4.3. Inter-Individual Variation

There was a significant inter-individual variation in the number of warnings and electric impulses the cows received in this study. Similar differences between individuals in relation to virtual fencing have also been shown in previous studies [8,12,14,19]. There was clear variation in how many warnings the cows received in the two periods, and how quickly they learned to avoid warnings and electric impulses. Additionally, there was a strong positive correlation between the number of warnings a cow received in each of the three respective periods. This clearly supports the hypothesis that the number of warnings and electric impulses are dependent on the individual cow. It is, therefore, suggested that the personality of each cow is taken into consideration when choosing individuals to be used in virtual fencing. However, this is only realisable in small scale projects. Clearly, it is desirable that cows, both for farming and nature management purposes, using virtual fencing, should have personalities suited to such circumstances [8,20]. This study clearly showed that cows receiving a small number of warnings in a training period will continue to receive few warnings and therefore electric impulses and will most likely be exposed to fewer stressful events.

It is also clear that in future investigations of animal welfare and learning capabilities of cattle, one needs to consider both the personality of each individual as well as socially facilitated behaviour. Statistical analyses should as such also take this into consideration, by assessing each individual in addition to pooled herd data. Furthermore, future studies should consider incorporating different breeds of cattle, and the effect of adding bulls to the herd should also be investigated. Another idea for future experimentation is to examine whether virtual fencing is effective in very large enclosures where the cattle would possibly not be subject to warnings or electric impulses for long periods of time. It may be more effective to create smaller virtual enclosures and move them, as the results indicate that cows are quick to adapt to new virtual borders, and previous studies show that virtual fencing is effective at herding cattle into a new area [36].

One aspect this experiment failed to address, is the possible link between personality and social hierarchy as well as possible socially facilitated learning. Previous studies have shown that cattle learned how to respond to virtual fencing, not just by interacting with the fence themselves, but also from observing herd mates [25,27]. Future studies are needed to investigate this potential link between social hierarchy and personality, possibly explaining why some individuals receive more auditory warnings and electric impulses than others, e.g., an individual receiving many warnings may be the dominant cow leading the herd. Future studies could also examine whether this social learning and hierarchical herd behaviour would allow for only a part of the herd to wear collars, without compromising the ability of the virtual fencing to contain the animals, as has been shown to be the case for sheep [26].

## 5. Conclusions

In conclusion, the Nofence virtual fencing system was effective at confining the herd of cattle to the desired area. No indications that virtual fencing negatively affected animal welfare were found based on the behavioural observations in this study. However, it is difficult to assess whether the welfare implications are larger or smaller compared to traditional electric fence, as no control group was included. Future studies should compare the two fencing methods to reach a conclusion. The animals did, however, show clear signs of learning, receiving fewer warnings and electrical impulses with time. As such, any welfare implications should be significantly reduced over time. Furthermore, the cows reacted to herd members receiving electric pulses, and future studies should not assume independency between the cows, but factor in herd behaviour, when doing statistical analyses. Lastly, the cows also expressed inter-individual variations in their responses to the virtual enclosure. This need to be taken into account when choosing animals to be placed in virtually fenced enclosures in order to ensure optimal welfare.

## Figures and Tables

**Table 1:** Overview and description of data collected for the data analysis with each message type sent from the Nofence© collars.

Type	Frequency	Description	No. of Obs.
*Poll *	Every 15 min	Positional data	157,120
*Seq*	Every 30 min	Activity data	79,633
*Warning*	After receiving warning	Positional data	1949
*Zap*	Upon receiving electric impulse	Positional data	197

**Figure A1:**
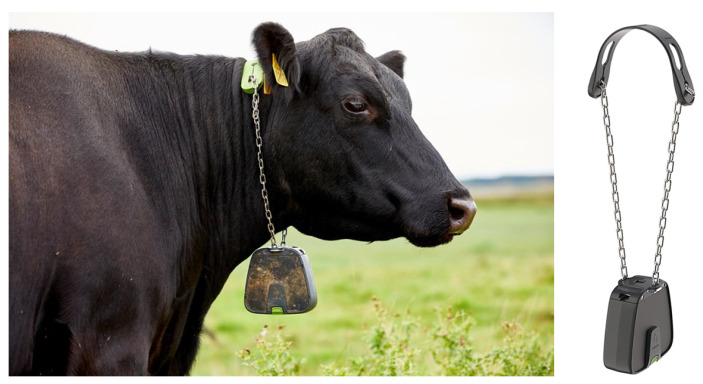
Nofence collar (Photo: J. Frikke).

**Figure A2:**
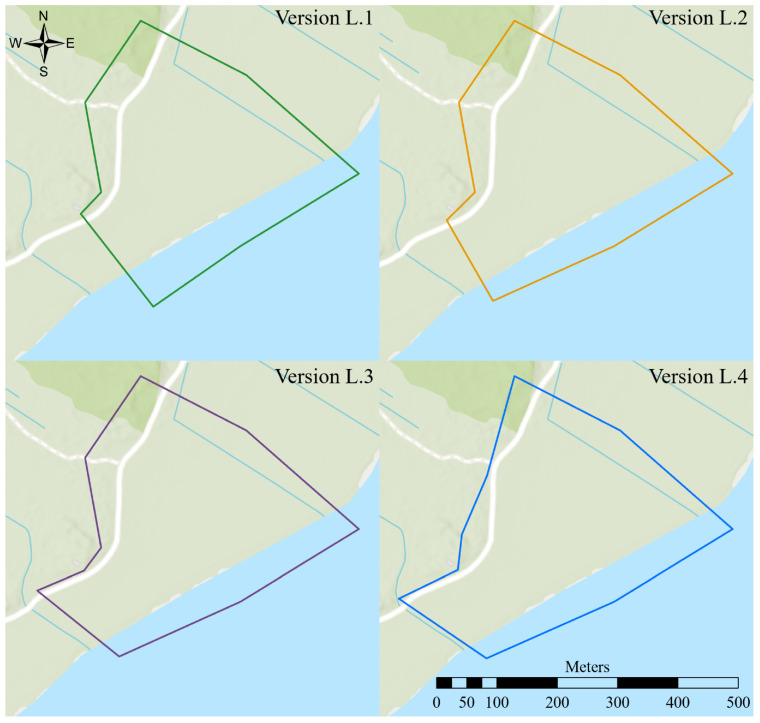
The virtual enclosure during *the learning period*. The coordinates of the lower right corner of each map frame are $8^{\circ }$27${}^{\prime }$59${}^{\prime \prime }$ E $55^{\circ }$24${}^{\prime }$4${}^{\prime \prime }$ N.

**Figure A3:**
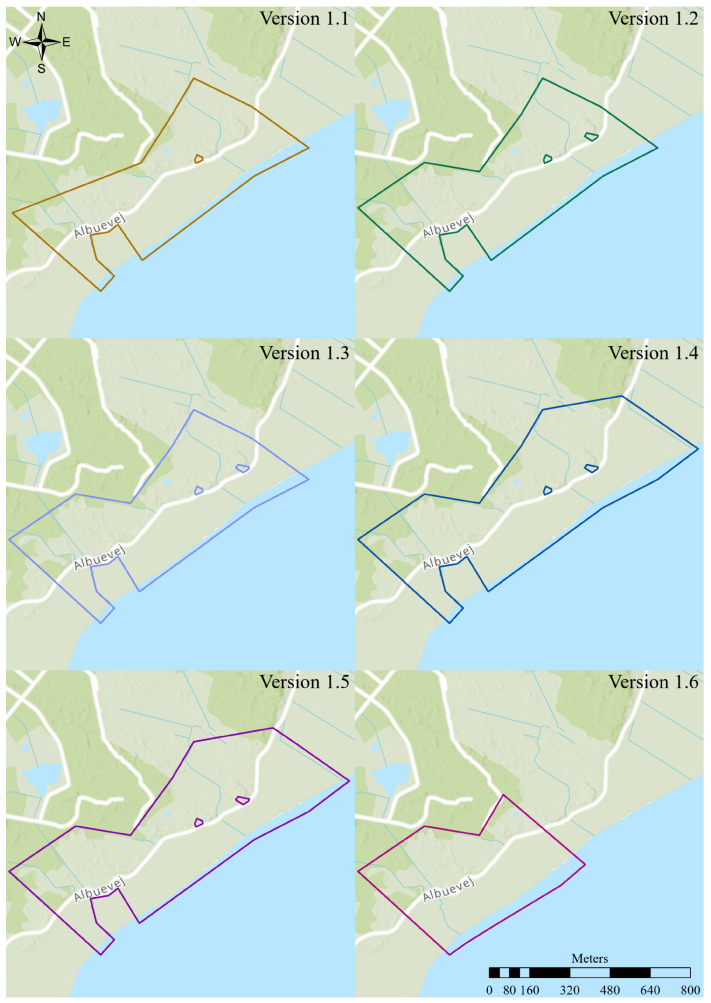
The virtual enclosure during *period 1*. The coordinates of the lower right corner of each map frame are $8^{\circ }$27${}^{\prime }$59${}^{\prime \prime }$ E $55^{\circ }$23${}^{\prime }$46${}^{\prime \prime }$ N.

**Figure A4:**
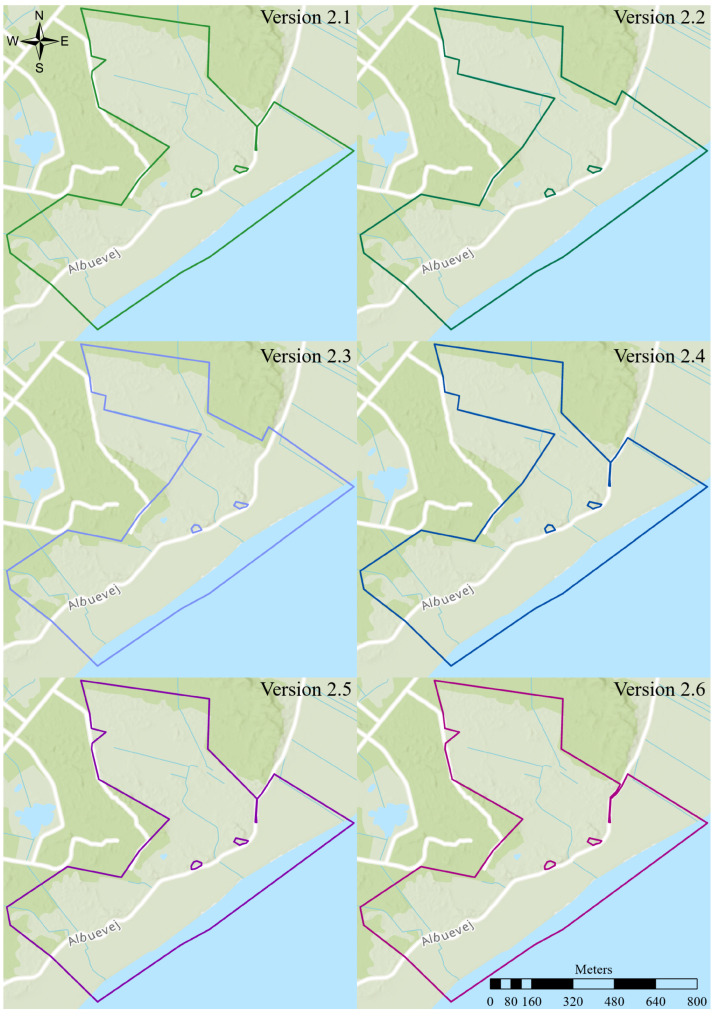
The virtual enclosure during *period 2*. The coordinates of the lower right corner of each map frame are $8^{\circ }$27${}^{\prime }$59${}^{\prime \prime }$ E $55^{\circ }$23${}^{\prime }$50${}^{\prime \prime }$ N.

**Figure A5:**
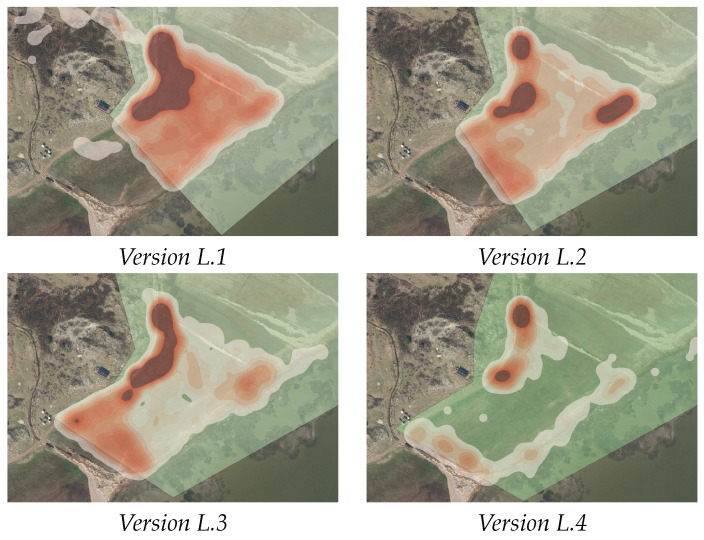
Heatmaps showing the cows’ distribution within the different versions of the virtual enclosure during the learning period. The light green area represents the area of the virtual enclosure for each version.

**Figure A6:**
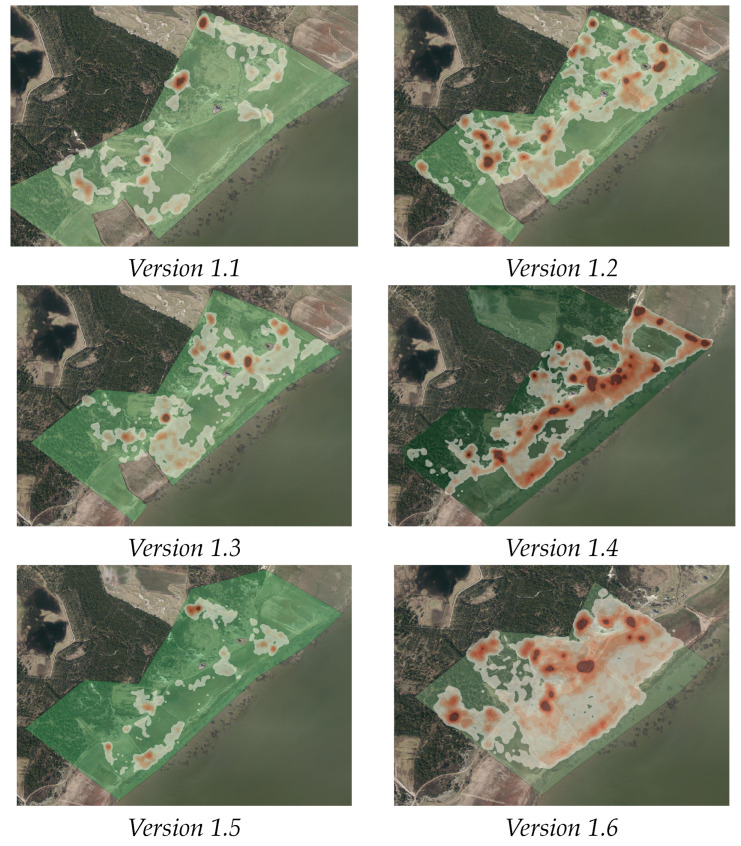
Heatmaps showing the cows’ distribution within the different versions of the virtual enclosure during period 1. The light green area represents the area of the virtual enclosure for each version.

**Figure A7:**
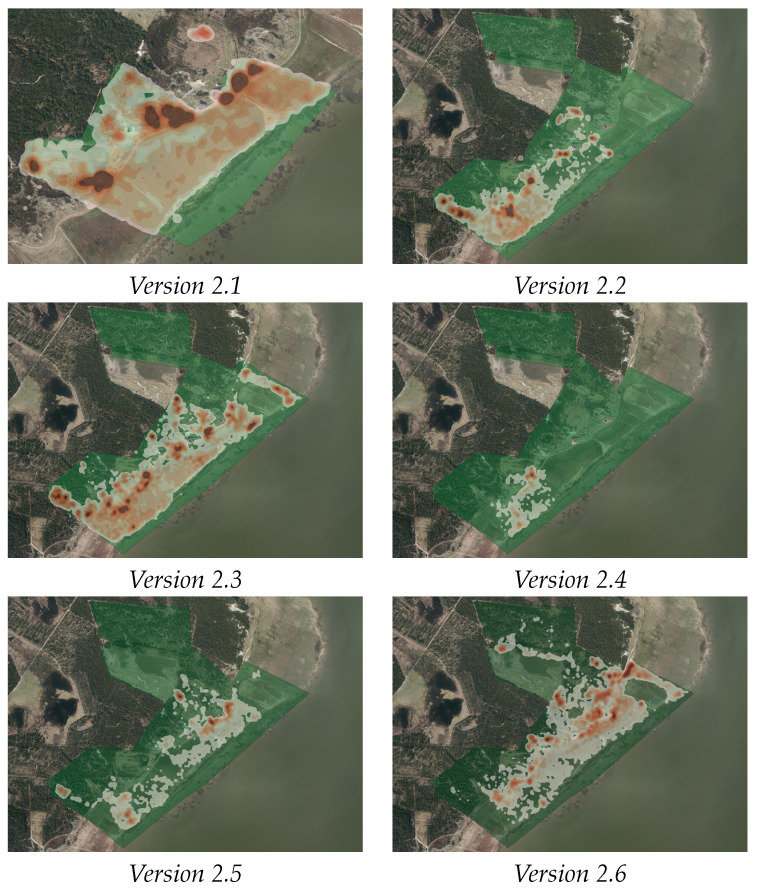
Heatmaps showing the cows’ distribution within the different versions of the virtual enclosure during period 2. The light green area represents the area of the virtual enclosure for each version.

**Figure A8:**
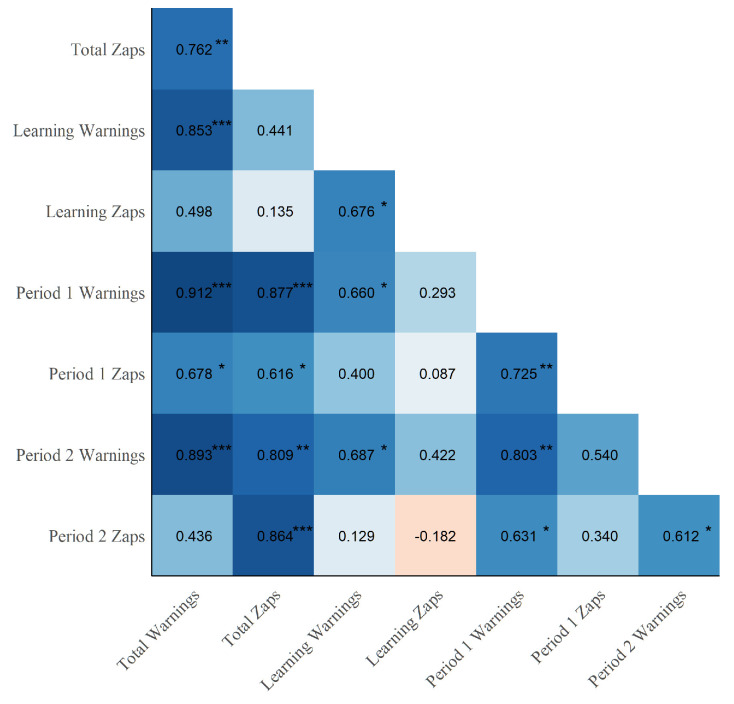
Correlation between auditory warnings and electric pulses received throughout the entire experiment and those received in each of the three periods. The values indicating the Spearman correlation coefficient with the significance level (* *p* < 0.05; ** *p* < 0.01; *** *p* < 0.001).

**Figure A9:**
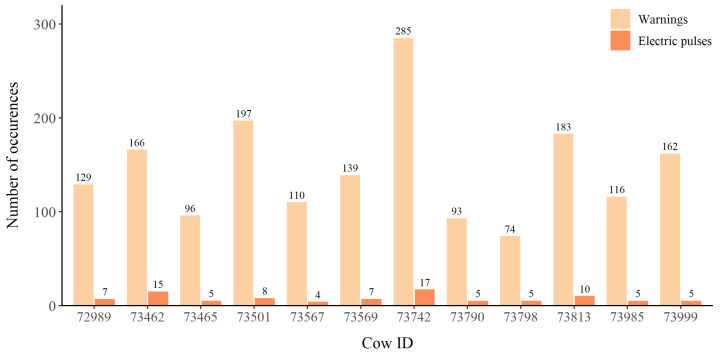
The number of auditory warnings and electric impulses that each individual received throughout the 139-day experiment, excluding those received on days where a social panic reaction occurred.

**Figure A10:**
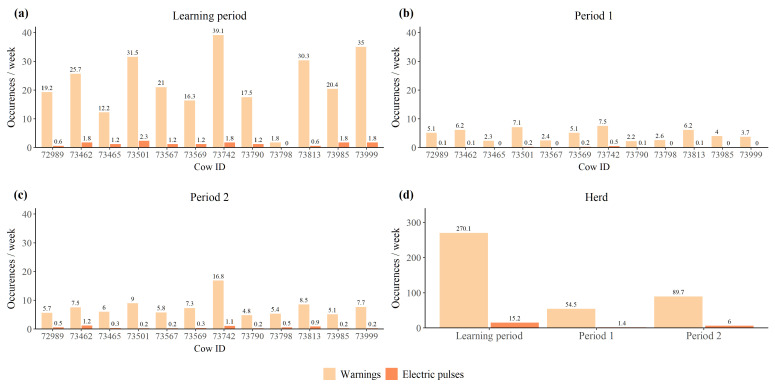
Average number of auditory warnings and electric impulses received per week by (**a**) each individual in *the learning period*, (**b**) each individual in *period 1*, (**c**) each individual in *period 2*, and (**d**) the entire herd during each of the three periods. Excluding days where a social panic reaction occurred.

**Figure A11:**
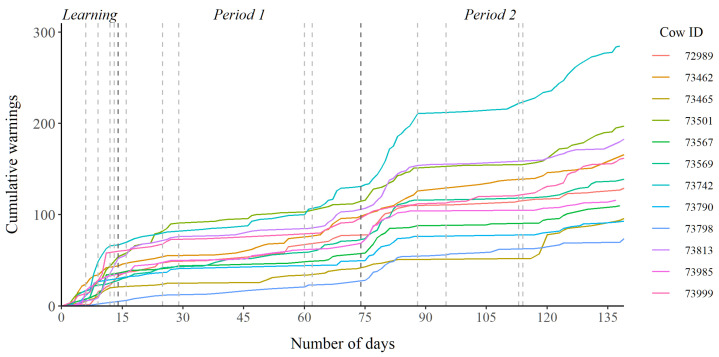
Cumulative number of warnings received by each cow during the three periods. The vertical lines in the plot mark any significant change in the virtual enclosure, where the darker lines indicate the division of the three periods. Excluding days where a social panic reaction occurred.

**Figure A12:**
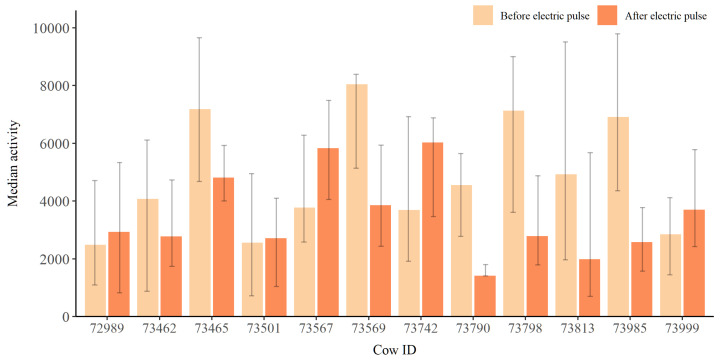
Median activity of each individual two hours before and after receiving an electric impulse (n=202). Error bars represent the interquartile range. An asterisk next to the serial number denotes statistical significance (*p* < 0.05). Excluding data from days where a social panic reaction occurred.

## Data Availability

The data presented in this study are available on request from the corresponding author.

## Appendix B. Virtual Enclosures

**Table A1:** Activation date and effective size of each version of the virtual enclosure. Each version of the enclosure was active from the date specified in the table until the next version was created. Only versions of the enclosure that were in effect for more than 12 h are included.

	Version	Date	Size (ha)
**Learning**	L.1	29 May 2021	11.92
	L.2	4 June 2021	12.08
	L.3	7 June 2021	12.55
	L.4	10 June 2021	12.26
**Period 1**	1.1	12 June 2021	34.94
	1.2	14 June 2021	37.41
	1.3	23 June 2021	37.23
	1.4	27 June 2021	45.80
	1.5	28 July 2021	48.93
	1.6	30 July 2021	27.98
**Period 2**	2.1	11 August 2021	15.08
	2.2	25 August 2021	64.72
	2.3	1 September 2021	64.80
	2.4	19 September 2021	63.03
	2.5	20 September 2021	69.50
	2.6	23 September 2021	70.36

## Appendix E. Pairwise Comparisons between Individuals

Results of pairwise comparisons between the individuals using the Mann-Whitney U Test with corrections for multiple testing using the Bonferroni correction.

**Table A2:** Individual differences in the number of auditory warnings and electric pulses they received throughout the entire experiment, the learning period, period 1, and period 2, respectively. The percentage of pairwise comparisons that showed significant differences between the individuals along with the significance level of the pairwise comparisons.

		Total Significant	No. Siginficant Differences			
Period	Message	Differences (%)	<0.05	<0.01	<0.001	n.s.
All	Warnings	70.0%	4	2	40	20
	Electric impulses	70.0%	4	2	40	20
Learning	Warnings	53.0%	3	4	28	31
	Electric impulses	9.10%	1	4	1	60
Period 1	Warnings	62.1%	2	6	33	25
	Electric impulses	51.5%	19	0	15	32
Period 2	Warnings	53.0%	2	6	27	31
	Electric impulses	22.7%	3	3	9	51

## Appendix F. Results when Removing the Social Panic Reaction Data

This appendix includes results where data from three days has been removed (29 May 2021; 10 June 2021; 20 September 2021). Two of these days all twelve cows escaped due to a social panic reaction, and the last day eight of the cows escaped due to a social panic reaction. Each time a cow escapes it has received three electric impulses before the system registers the cow as ’escaped’.

### Appendix F.2. Learning Ability

The cows received significantly more auditory warnings and electric impulses per week during the 14 day *learning period* than during the other two periods (*p* < 0.05) (Figure A10). The cows also received significantly more warnings and electric impulses in *period 2* compared to *period 1* (*p* < 0.05) (Figure A10). Moreover, the ratio between the number of electric impulses and the number of auditory warnings was highest in *the learning period* for most of the individuals and all individuals had the lowest impulse to warning ratio in *period 1*.

### Appendix F.3. Inter-Individual Differences

There was a positive correlation between the number of warnings and electric impulses an individual received over the entire period of the experiment ($r_{s}=0.817$ **). There was also a positive correlation between the number of warnings an individual cow received in *the learning period* and the number of warnings received in *period 1* ($r_{s}=0.649$ *) and *period 2* ($r_{s}=0.748$ **), respectively. Furthermore, the individuals significantly differed from one another in the number of warnings and electric impulses they received (*p* < 0.001).

### Appendix F.4. Reactions to Electric Impulse

There were no significant changes in the cows’ activity before and after receiving an electric impulse (Figure A12).
